# Patient Stratification for Serum LDH Levels Reveals Distinct CLA^+^ T-Cell Cytokine Secretion in Response to HDM, Clinical Features and Allergic Comorbidities

**DOI:** 10.3390/ijms26167821

**Published:** 2025-08-13

**Authors:** Irene García-Jiménez, Ignasi Figueras-Nart, Lídia Sans-de San Nicolás, Laia Curto-Barredo, Marta Bertolín-Colilla, Montserrat Bonfill-Ortí, Sandra Díez-Ribas, Alex Llobet-del Pino, Antonio Guilabert-Vidal, Anna Ryzhkova, Marta Ferran, Ramon M. Pujol, Luis F. Santamaria-Babí

**Affiliations:** 1 Immunologia Translacional, Departament de Biologia Cellular, Fisiologia i Immunologia, Facultat de Biologia, Universitat de Barcelona (UB), Parc Científic de Barcelona (PCB), 08028 Barcelona, Spain; irenegarciajimenez@ub.edu (I.G.-J.);; 2 Programa de Doctorat en Biomedicina, Universitat de Barcelona (UB), 08028 Barcelona, Spain; 3 Departament de Dermatologia, Hospital de Bellvitge, Universitat de Barcelona (UB), 08907 L’Hospitalet de Llobregat, Spain; 4 Departament de Dermatologia, Hospital del Mar, Institut Hospital del Mar d’Investigacions Mèdiques (IMIM), Universitat Autònoma de Barcelona (UAB), 08003 Barcelona, Spain; 5 Departament de Dermatologia, Hospital General de Granollers, 08402 Granollers, Spain

**Keywords:** atopic dermatitis, allergic comorbidities, cutaneous lymphocyte-associated antigen, house dust mite, lactate dehydrogenase, patient stratification

## Abstract

Lactate dehydrogenase (LDH) is a serum biomarker well known to correlate with disease severity in atopic dermatitis (AD). The aim of this study was to explore the cutaneous immune responses and the clinical profile of AD patients in relation to serum LDH levels. To this end, 47 untreated, adult patients with moderate-to-severe AD were stratified by median levels of serum LDH. Circulating memory T-cell responses to house dust mite (HDM) extract, in the presence of autologous lesional epidermal cells, were compared between AD subgroups. The LDH^high^ group exhibited significantly higher IL-13, IL-5 and IL-9 in vitro responses confined to the cutaneous lymphocyte-associated antigen (CLA)^+^ subset compared to LDH^low^ patients. Clinically, LDH^high^ patients were younger and exhibited more severe disease, elevated eosinophil counts in their blood, increased total and specific IgE levels in their plasma, and a higher prevalence of allergic rhinitis. Our data suggests that high LDH levels identify a subgroup of AD patients with a specific immune and clinical profile, and highlight the potential of LDH as a clinical parameter that may enable patient stratification for treatment selection.

## 1. Introduction

Atopic dermatitis (AD) is a common, heterogeneous, chronic inflammatory, T-cell-mediated cutaneous disease affecting up to 10% of adults worldwide. This disorder is characterized by a dysfunctional epidermal barrier, an altered skin microbiome composition, an abnormal immune response and intense itching. Allergic comorbidities, including allergic asthma, rhinitis, conjunctivitis, eosinophilic esophagitis and food allergy, frequently co-occur with atopic dermatitis [1,2].

Lactate dehydrogenase (LDH) is an enzyme involved in the metabolism of carbohydrates present in multiple tissues, including skin. LDH is increased in several cutaneous diseases, including atopic dermatitis, as first reported by Mukai H et al. in 1990 [3,4]. They observed increased LDH levels in patients with moderate-to-severe AD, and since then, numerous studies have reported serum LDH to strongly and significantly correlate with AD disease severity [4,5]. In addition, LDH seems to reflect skin barrier dysfunction, as it is directly correlated with barrier integrity—transepidermal water loss measurement—and inversely with skin hydration [6]. Serum LDH also correlates with circulating eosinophil counts and serum immunoglobulin (Ig)E levels [4,7,8], and has been found to be elevated in moderate-to-severe AD patients colonized by *Staphylococcus aureus* (*S. aureus*) compared to non-colonized patients [6].

Circulating cutaneous lymphocyte-associated antigen (CLA)^+^ T cells have emerged as valuable peripheral cellular biomarkers, since they reflect cutaneous abnormalities present in AD skin [9]. Interestingly, treatment with the specific T helper 2 (Th2)-targeting agents dupilumab and tralokinumab has been shown to influence the proliferation and cytokine production of circulating CLA^+^ T cells, highlighting potential CLA^+^ T-cell responses to gain insights into treatment efficacy in AD patients [10,11,12,13]. Directed therapies against the co-stimulatory T-cell receptor OX40 and its ligand, OX40L, with the former molecule being particularly elevated in circulating CLA^+^ T cells in patients with AD [14], offer promising new treatment options [15]. Of note, evaluating the effector function of CLA^+^ T cells activated by clinically relevant triggers of AD has allowed the stratification of patients based on the production of interleukin (IL)-9, IL-13 and IL-31 and in relation to their clinical status [16,17,18].

In the present study, we analyzed the different allergen-specific CLA^+^ T-cell effector functions, clinical features and allergic comorbidities of AD patients stratified by serum LDH level. We found that AD patients with high serum LDH display increased production of IL-13, IL-5 and IL-9 cytokines by CLA^+^ memory T cells in response to the house dust mite (HDM) allergen and have a more severe disease, accompanied by more allergic comorbidities, compared to patients with low LDH levels.

## 2. Results

### 2.1. Patients with High Serum LDH Show Higher IL-13, IL-5 and IL-9 Production by CLA^+^ Memory T Cells in Respose to HDM than Patients with Low Serum LDH

**Figure 1:**
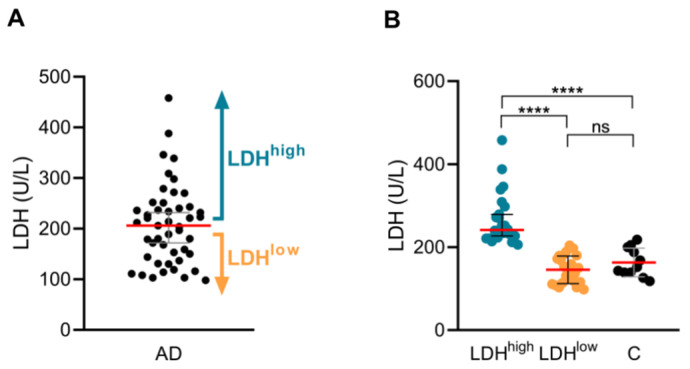
**Serum LDH levels define LDH^high^ and LDH^low^ AD subgroups.** (**A**,**B**) Serum LDH levels in LDH^high^ (*n* = 24) and LDH^low^ (*n* = 23) AD patients, and control subjects (*n* = 11). AD, atopic dermatitis; C, control subjects; LDH, lactate dehydrogenase; LDH^high^, AD patients with high serum LDH levels; LDH^low^, AD patients with low serum LDH levels. ns: *p* > 0.05; **** *p* < 0.0001.

Serum LDH levels were assessed in 47 untreated patients with moderate-to-severe AD. Patients were stratified into LDH^high^ (≥206 kU/L, *n* = 24) and LDH^low^ (<206 kU/L, *n* = 23) groups based on median serum LDH levels (Figure 1A), with the LDH^low^ group remaining within the control range (Figure 1B).

**Figure 2:**
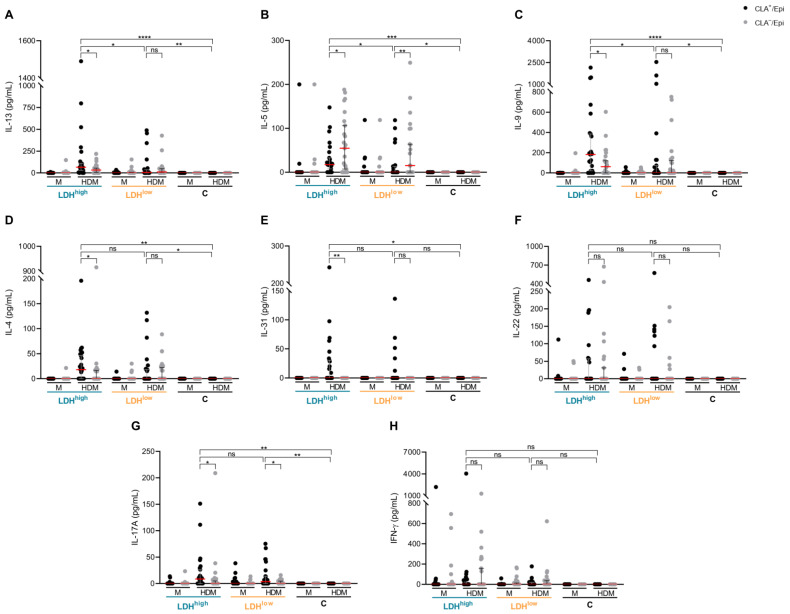
**LDH^high^ patients have the highest IL-13, IL-5 and IL-9 production by CLA^+^ memory T cells in response to the HDM allergen.** (**A**–**H**) The levels of IL-13, IL-5, IL-9, IL-4, IL-31, IL-22, IL-17A and IFN-γ (pg/mL) produced by HDM-induced CLA^+^ T-cell cocultures were compared between LDH^high^ patients (*n* = 23), LDH^low^ (*n* = 23) patients and controls (*n* = 11). C, control subjects; CLA, cutaneous lymphocyte-associated antigen; Epi, epidermal cell suspension; HDM, house dust mite; IFN, interferon; IL, interleukin; LDH, lactate dehydrogenase; LDH^high^, AD patients with high serum LDH levels; LDH^low^, AD patients with low serum LDH levels; M, untreated. ns: *p* > 0.05; * *p* < 0.05; ** *p* < 0.01; *** *p* < 0.001; **** *p* < 0.0001.

To evaluate the response of circulating skin-tropic CLA^+^ and systemic/non-cutaneous CLA^−^ memory T cells, they were cocultured with autologous lesional epidermal cells and activated with HDM extract. The production levels of IL-13, IL-5 and IL-9, but not IL-4, IL-17A, IL-22, IL-31 and interferon (IFN)-γ, were found to be more elevated in CLA^+^ T-cell cocultures from LDH^high^ patients compared to LDH^low^ patients, revealing a Th2-skewed response in the former group (Figure 2). Conversely, a similar cytokine response was observed in CLA^−^ T-cell cocultures from both AD subgroups. Interestingly, no cytokine secretion was observed in CLA^+^ or CLA^−^ T-cell cocultures from control individuals when directly challenged with HDM extract.

### 2.2. Patients with High Serum LDH Present a Specific Phenotype and a Higher Prevalence of Allergic Comorbidities in Comparison with Patients with Low Serum LDH

**Figure 3:**
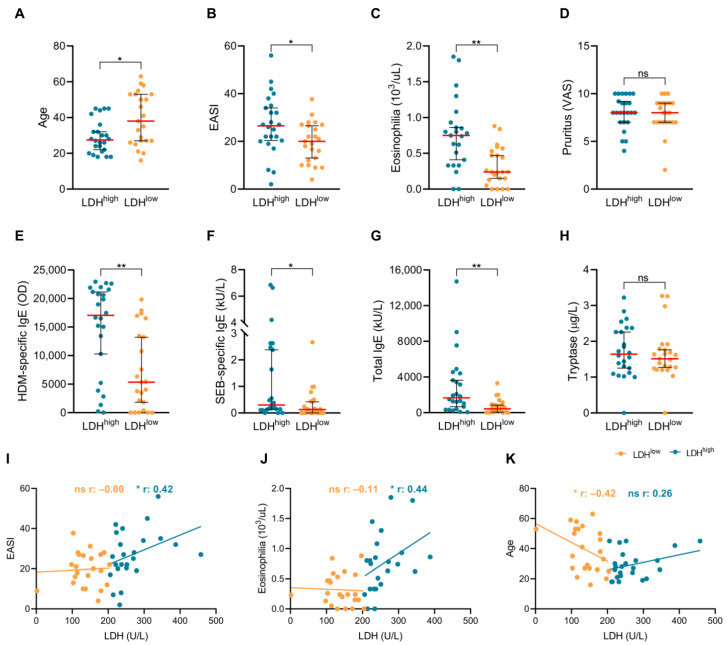
**LDH^high^ patients are younger and have more severe disease, elevated eosinophilia, increased sensitization status and a higher prevalence of allergic comorbidities.** (**A**) Age, (**B**) EASI, (**C**) eosinophilia, (**D**) pruritus, (**E**) HDM-specific IgE, (**F**) SEB-specific IgE, (**G**) total IgE and (**H**) tryptase were compared between groups (*n* = 24 for LDH^high^ and *n* = 22–23 for LDH^low^). Serum LDH levels were correlated with (**I**) EASI, (**J**) eosinophilia and (**K**) age in LDH^high^ (*n* = 24) and LDH^low^ (*n* = 22–23) patients. AD, atopic dermatitis; EASI, eczema area and severity index; HDM, house dust mite; LDH, lactate dehydrogenase; LDH^high^, AD patients with high serum LDH levels; LDH^low^, AD patients with low serum LDH levels; OD, optical density; SEB, staphylococcal enterotoxin B; VAS, visual analogue scale. ns: *p* > 0.05; * *p* < 0.05; ** *p* < 0.01.

We explored whether LDH^high^ and LDH^low^ patients differed clinically. We found that LDH^high^ patients were younger, and they exhibited increased disease severity and eosinophil counts in their blood than the LDH^low^ subgroup (Figure 3A–C). Interestingly, serum LDH levels directly correlated with disease severity and eosinophil count exclusively in LDH^high^ patients, and not in LDH^low^ individuals (Figure 3I,J). Contrarily, a strong indirect correlation between serum LDH levels and age was observed for LDH^low^ patients, whereas a tendency toward a positive correlation was observed in the LDH^high^ subgroup (Figure 3K). Furthermore, although LDH^high^ subjects presented elevated HDM- and staphylococcal enterotoxin B (SEB)-specific and total IgE levels in their plasma compared to LDH^low^ patients (Figure 3E–G), the extent of patients’ HDM and SEB sensitization and their overall allergic status were significantly heightened in both AD subgroups in comparison with the controls (Table S1). No differences in gender, pruritus intensity or tryptase plasma levels were observed between the AD subgroups (Table 1, Figure 3D,H).

Allergic rhinitis, conjunctivitis and asthma, along with food allergy, are common conditions of the atopic spectrum that frequently co-occur with atopic dermatitis, especially in moderate-to-severe forms [19]. Next, we explored the frequency of these allergic comorbidities within the LDH^high^ and LDH^low^ subgroups and observed a significantly higher prevalence of allergic rhinitis and an increased tendency to exhibit allergic conjunctivitis, but not asthma or food allergy, in the former AD subgroup (Table 1).

### 2.3. Stratification According to Disease Severity, Eosinophil Count or Serum Total IgE in the Same Patients Fails to Reflect LDH^high^ Endotype

Since total IgE, circulating eosinophil count and disease severity strongly correlate with serum LDH in AD [4,5,20,21], we explored whether stratifying the same cohort of patients based on the median levels of these clinical parameters may reveal AD subgroups with similar CLA^+^ T-cell-mediated cytokine responses as defined by serum LDH classification.

**Figure 4:**
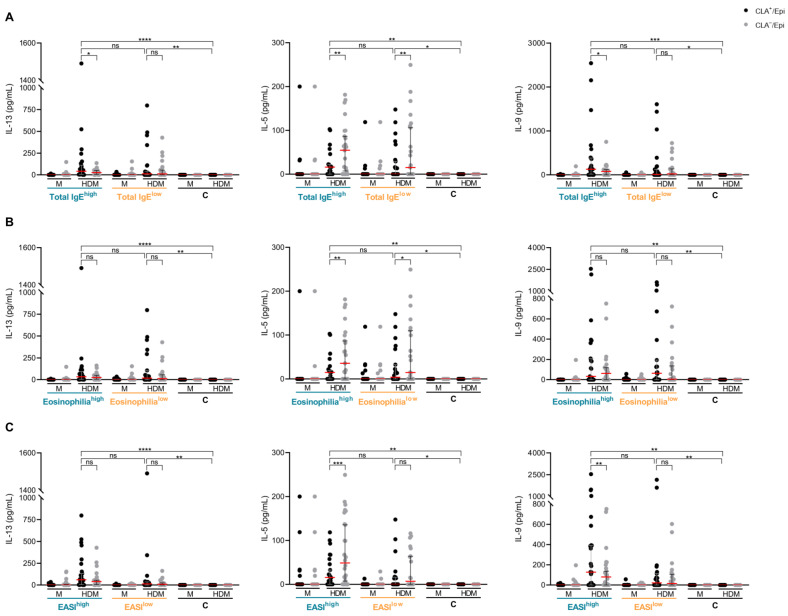
**Comparison of IL-13, IL-5 and IL-9 production levels between AD subgroups, defined by total IgE, eosinophilia or EASI.** Levels of IL-13, IL-5 and IL-9 (pg/mL) produced by HDM-induced CLA^+^ T-cell cocultures were compared between AD subgroups, defined by median levels of (**A**) total IgE (Total IgE^high^ (*n* = 23) and Total IgE^low^ (*n* = 23)), (**B**) eosinophilia (Eosinophilia^high^ (*n* = 23) and Eosinophilia^low^ (*n* = 22)) and (**C**) EASI (EASI^high^ (*n* = 26) and EASI^low^ (*n* = 20)). C, control subjects; CLA, cutaneous lymphocyte-associated antigen; EASI, eczema area and severity index; EASI^high^, AD patients with high disease severity; EASI^low^, AD patients with low disease severity; Eosinophilia^high^, AD patients with high eosinophil counts in blood; Eosinophilia^low^, AD patients with low eosinophil counts in blood; Epi, epidermal cell suspension; HDM, house dust mite; IL, interleukin; LDH, lactate dehydrogenase; M, untreated; Total IgE^high^, AD patients with high total IgE levels in plasma; Total IgE^low^, AD patients with low total IgE levels in plasma. ns: *p* > 0.05; * *p* < 0.05; ** *p* < 0.01; *** *p* < 0.001; **** *p* < 0.0001.

To explore this hypothesis, AD patients were first divided into Total IgE^high^ (≥844.9 kU/L, *n* = 24) and Total IgE^low^ (<844.9 kU/L, *n* = 23) groups (Figure S1A). Next, patients were classified, according to the median counts of circulating eosinophils, into Eosinophilia^high^ (≥0.47 × 10^3^/μL, *n* = 24) and Eosinophilia^low^ (<0.47 × 10^3^/μL, *n* = 22) groups (Figure S1B). Finally, AD patients, adjusted for disease severity, were classified into eczema area and severity index (EASI)^high^ (≥22, *n* = 27) and EASI^low^ (<22, *n* = 20) groups based on the median scores (Figure S1C). None of these stratifications indicated differential secretion of IL-13, IL-5 and IL-9 by CLA^+^ memory T cells in response to HDM between the resulting AD subgroups (Figure 4).

In addition, to further evaluate whether the observed differences in IL-13, IL-5 and IL-9 levels between LDH^high^ and LDH^low^ groups (Figure 2A–C) were independent of disease severity, we performed median regression analyses adjusting for EASI scores. These analyses confirmed that patients with elevated serum LDH exhibited heightened IL-5 and IL-9 responses independently of disease severity, and IL-13 responses showed a similar trend (Table S2).

Interestingly, the prevalence of allergic comorbidities was similar among subgroups, independently of the stratification criteria used (Table 2).

## 3. Discussion

Serum LDH is a reliable biomarker demonstrated by numerous studies to correlate with disease severity in AD [5]. However, its potential to functionally stratify patients with moderate-to-severe AD has not been previously explored. Our study shows that classifying patients according to high and low serum LDH allows the identification of a subgroup of AD patients with a distinct CLA^+^ T-cell effector response and clinical profile.

We studied the HDM response in an ex vivo model of AD—based on the coculture of circulating memory T cells and autologous lesional epidermal cells—that has been demonstrated to generate valuable information by establishing a relationship between cytokine levels secreted by CLA^+^ T cells and the clinical profile of patients [16,17,18]. We found that the secretion of IL-13, IL-5 and IL-9 by CLA^+^ memory T cells in response to HDM extract was significantly higher in LDH^high^ patients compared with LDH^low^ patients.

IL-13 is a central pathogenic mediator in AD, since there is dominant expression of this cytokine over IL-4 in lesional AD skin [22], which is abundantly infiltrated by CLA^+^ T cells [23,24]. Acevedo N et al. found that reduced DNA methylation in the upstream region of the IL13 gene in CD4^+^ CLA^+^ T cells from AD patients with a severe phenotype led to an augmented ability of CLA^+^ T cells to secrete IL-13 [24]. Accordingly, we show higher IL-13 secretion by CLA^+^ memory T cells from LDH^high^, a subgroup with a more severe disease. The substantial production of IL-13 may be due to the higher severity of LDH^high^ patients, consistent with previous reports indicating that the frequency of IL-13-producing CLA^+^ T cells is directly associated with disease severity [25]. Importantly, the reduction in circulating CLA^+^ T cells producing IL-13 reflects the early treatment effect of dupilumab and tralokinumab [13,26], clinically supporting the central role of CLA^+^ T-cell-mediated IL-13 secretion in the pathophysiology of the disease.

Interestingly, a recent study demonstrated that serum LDH levels significantly dropped in AD patients after 3 months of dupilumab treatment, and found a positive correlation between the reduction in EASI scores and serum LDH levels [27]. Accordingly, Lee Y et al. observed a decline in serum LDH levels after 16 weeks of dupilumab therapy, but they also noticed that EASI-75 response was significantly more frequent in patients with serum LDH levels < 400 U/L [28]. These studies seem to indicate that serum LDH levels are somewhat related to dupilumab response in patients with AD. However, data is limited, and further studies are needed to confirm this notion.

Consistent with the known role of IL-13 in driving IgE class switching [29], we observed elevated total and specific IgE levels in LDH^high^ patients. Furthermore, IL-13 is involved in skin barrier dysfunction by decreasing the expression of key structural proteins [30]. A compromised barrier can facilitate *S. aureus* colonization and allergen penetration in AD skin. Indeed, AD patients colonized by *S. aureus* showed greater allergen sensitization, barrier dysfunction and serum LDH levels than non-colonized patients [6]. These findings align with the increased IL-13 levels and marked sensitization to HDM and SEB observed in LDH^high^ patients, and support the functional stratification of AD patients into LDH^high^ and LDH^low^ subgroups.

IL-5 secretion was especially high in LDH^high^ patients, which could be related to the increased eosinophil count in their blood and may explain the higher prevalence of allergic rhinitis within this AD subpopulation. Importantly, significantly more IL-5 was produced by CLA^–^ than the CLA^+^ T-cell population upon HDM activation. In fact, previous studies have shown that HDM extract preferentially expands the CLA^–^ fraction in asthmatic patients [31]. This systemic response may contribute to the development of allergic comorbidities by promoting inflammation in non-cutaneous organs. While specific IL-5-targeting agents have failed to achieve meaningful improvement in AD [32], they have been shown to reduce eosinophil counts and ameliorate allergic rhinitis symptoms in asthma [33,34].

IL-9 is involved in allergy and atopic dermatitis [18]. Some studies have shown that IL-9 enhances IL-4-mediated IgE production [35,36]. Accordingly, we show elevated total and specific IgE levels in LDH^high^ patients, which may be due to the elevated IL-9 levels secreted by CLA^+^ memory T cells. Our observations are supported by previously published data from our group showing increased LDH levels in patients with increased IL-9 production in response to HDM extract [18]. Despite the discontinuation of omalizumab due to lack of benefit in severe AD [32], treatment with this anti-IgE mAb led to reduced IL-9 plasma levels in pediatric AD [37], contributing to evidence for the link between IL-9 and IgE isotype switching in this condition.

Taken together, finding that HDM-induced CLA^+^ memory T-cell production levels of IL-13, IL-5 and IL-9 are linked with the clinical features of LDH^high^ patients is of translational relevance, since a recent report—in which the researchers performed TCR sequencing—demonstrated that allergen-specific CLA^+^ T-cell clones infiltrating AD skin lesions correspond to the circulating CLA^+^ T cells found in peripheral blood [38].

The classification of our cohort of patients based on the median levels of serum LDH showed that LDH^high^ patients were younger and had more severe disease, increased eosinophil counts in their peripheral blood, elevated total and specific IgE levels in response to HDM and SEB, and a higher prevalence of allergic comorbidities. In addition, we found that serum LDH only correlated with disease severity and eosinophilia in the LDH^high^ group, possibly reflecting the lower severity in LDHl^ow^ patients. These findings indicate that LDH^high^ and LDH^low^ patients are clinically distinct, except for pruritus and the levels of tryptase in their plasma, suggesting the potential utility of serum LDH in stratifying AD patients. The lack of difference in pruritus intensity between AD subgroups may be due to their secretion of comparable levels of IL-4, IL-22 and IL-31, as well as similar levels of tryptase. Among other pruritogens, these mediators promote non-histaminergic itching [39,40,41], and the blockade of IL-4, IL-22 and IL-31 improves pruritus in AD [42,43,44,45].

Given the strong correlation between serum LDH levels and total IgE, blood eosinophil counts and disease severity [4,5,20,21], it could be possible that the AD subgroups defined in the present study may not be specific to serum LDH levels, and that adjustment for any of those clinical parameters would provide similar outcomes. To test this hypothesis, the same cohort of patients was stratified according to median levels of either total IgE, blood eosinophil count or EASI. However, no differences in IL-13, IL-5 and IL-9 secretion by CLA^+^ memory T cells upon HDM activation were found between subgroups. Moreover, no higher prevalence of allergic rhinitis was observed when these alternative stratifications were applied. These observations reinforce serum LDH levels as a valuable tool to identify patients with a distinct immunological and clinical profile.

Although CLA^+^ memory T cells from LDH^high^ secreted more IL-13, IL-5 and IL-9 in response to HDM in vitro than those from LDH^low^ patients, both AD subgroups responded to HDM and produced significantly more IL-4, IL-5, IL-9, IL-13, IL-17A and IFN-γ—and IL-22 and IL-31 only in the LDH^high^ group—compared to controls. In addition to the distinct clinical features between LDH^high^ and LDH^low^ patients, the latter subgroup—despite having serum LDH levels within the control ranges—showed higher levels of total and specific IgE than the controls. These findings indicate that LDH^low^ patients exhibit a separated immunological and clinical profile in comparison to LDH^high^ and control subjects.

Our study has several limitations. Firstly, we acknowledge the arbitrary nature of the LDH cutoff as a limitation of the study. Secondly, the CLA^+^ T-cell response and the clinical features associated with high LDH levels in serum deserve to be further validated in a larger independent cohort of AD patients. Thirdly, the inclusion of sex-and age-matched controls remains a limitation of the study and should be considered in future study designs. Additionally, our findings are based on an adult Spanish cohort, and the observations may not generalize to pediatric populations or other ethnic backgrounds. Lastly, the presence of autoimmune diseases in patients and controls was not recorded. These potential confounding factors play an important role in the onset and progression of the disease [46] and may have influenced our findings.

In conclusion, we show, for the first time, that depending on serum LDH levels, circulating CLA^+^ T cells secrete a concrete cytokine profile that associates differently with the clinical features of each AD group.

## 4. Materials and Methods

### 4.1. Patients

This study included 47 consenting adult patients with moderate-to-severe AD and 11 non-age- and sex-matched consenting controls under institutional review board-approved protocols at Hospital General de Granollers and Hospital del Mar (Spain). The exclusion criteria included any topical or systemic anti-inflammatory treatments administered for a minimum of 2–4 weeks prior to the study. The clinical data of the AD patients and control subjects are summarized in Table S3.

### 4.2. Isolation of Circulating CLA^+/−^ Memory T Cells and Epidermal Cell Suspension

Peripheral blood mononuclear cells (PBLs) were isolated from whole blood by a Ficoll (GE Healthcare, Princeton, NJ, USA) gradient, and circulating central and effector memory CD45RA^−^ CLA^+^ and CLA^−^ T lymphocytes were purified using three consecutive immunomagnetic separations (Miltenyi Biotech, Bergisch Gladbach, Germany), as previously described [47].

Two skin punch biopsies of an active lesion were incubated in dispase (Corning, Corning, NY, USA) overnight at 4 °C. The epidermal sheet was peeled off from the dermis, cut into pieces and incubated with trypsin (Biological Industries, Kibbutz Beit Haemek, Israel) for 15 min at 37 °C. The epidermal tissue was mechanically disaggregated by pipetting, and the cell suspension (Epi) was transferred to fresh culture medium, as previously described.

### 4.3. Coculture and Stimulation of Circulating CLA^+/−^ Memory T Cells with Epidermal Cells

The ex vivo cocultures consisted of 5 × 10^4^ circulating CLA^+/−^ memory T cells and 3 × 10^4^ autologous lesional epidermal cells (CLA^+^/Epi or CLA^−^/Epi, respectively) in 96-well U-bottom plates (Falcon, Corning, Corning, NY, USA). The cocultures were left untreated (M) or stimulated with HDM extract (kindly provided by LETI Pharma, Barcelona, Spain) at a final well concentration of 10 μg/mL for 5 days,. Supernatants were collected and kept at −20 °C for later cytokine quantification.

### 4.4. Cytokine and Chemokine Quantification

A ProcartaPlex multiplex immunoassay (Invitrogen, Waltham, MA, USA) was conducted to measure IL-4, IL-5, IL-9, IL-13, IL-17A, IL-22, IL-31 and IFN-γ concentrations in the collected culture supernatants with a MAGPIX plate reader (Luminex Technologies Inc., Austin, TX, USA). The data was analyzed with ProcartaPlex Analyst software version 1.0 (Invitrogen) using a five-parameter logistic curve. Values below the lower limit of quantification (LLOQ) were treated as zero.

### 4.5. Quantification of Total and Specific IgE Against HDM and SEB

Total IgE (kU/L), HDM-specific IgE (response (OD)) and SEB-specific IgE (kU/L) plasma levels were measured by ImmunoCAP (ThermoFisher Scientific, Waltham, MA, USA).

### 4.6. Statistical Analysis

The data are presented as scatter dot plots and the median ± 95% confidence interval (CI). When comparing data between the LDH^high^ and LDH^low^ groups, for continuous variables, the sample median (25th–75th percentiles) was shown, and Mann–Whitney or Wilcoxon tests were used for differences between two groups or within the same group, respectively. Multiple testing correction was applied to pairwise comparisons with the Benjamini and Hochberg false discovery rate (FDR) method. For categorical variables, the raw numbers (percentages) were shown, and Fisher’s exact test was used. Values in bold indicate statistical significance. Correlations were represented as linear regressions, and the Spearman correlation coefficient was used. Statistical analyses and data presentation were conducted with GraphPad Prism software version 8 (GraphPad Software Corporation, San Diego, CA, USA). A *p*-value of less than 0.05 was considered significant.

## Figures and Tables

**Table 1:** Differences in categorical clinical features between LDH^high^ and LDH^low^.

Clinical Features	LDH^high^, *n* (%)	N	LDH^low^, *n* (%)	N	*p* Value
Female	10 (41.7%)	24	13 (56.5%)	23	0.614
Rhinitis	19 (79.2%)	24	9 (39.1%)	23	**0.039**
Conjunctivitis	13 (54.2%)	24	5 (21.7%)	23	0.089
Asthma	11 (45.8%)	24	11 (47.8%)	23	>0.999
Food allergy	6 (27.3%)	22	4 (17.4%)	23	0.614

Categorical variables are presented as counts (percentages). LDH, lactate dehydrogenase. Bold values indicate significant data.

**Table 2:** Distribution of allergic comorbidities within AD subgroups based on median levels of total IgE, blood eosinophil count and EASI.

Clinical Features	Total IgE^high^, *n* (%)	N	Total IgE^low^, *n* (%)	N	*p* Value
Rhinitis	17 (70.8%)	24	11 (47.8%)	23	0.285
Conjunctivitis	12 (50.0%)	24	6 (26.1%)	23	0.285
Asthma	13 (54.2%)	24	9 (39.1%)	23	0.514
Food allergy	4 (18.2%)	22	6 (26.1%)	23	0.722
**Clinical Features**	**Eosinophilia^high^, *n* (%)**	**N**	**Eosinophilia^low^, *n* (%)**	**N**	***p* Value**
Rhinitis	18 (75.0%)	24	10 (45.5%)	22	0.277
Conjunctivitis	12 (50.0%)	24	6 (27.3%)	22	0.281
Asthma	13 (54.2%)	24	8 (36.4%)	22	0.337
Food allergy	4 (17.4%)	20	6 (27.3%)	20	0.491
**Clinical Features**	**EASI^high^, *n* (%)**	**N**	**EASI^low^, *n* (%)**	**N**	***p* Value**
Rhinitis	17 (63.0%)	27	11 (55.0%)	20	0.765
Conjunctivitis	13 (48.2%)	27	5 (25.0%)	20	0.273
Asthma	14 (51.9%)	27	8 (40.0%)	20	0.742
Food allergy	9 (36.0%)	25	1 (5.0%)	20	0.108

Categorical variables are presented as counts (percentages). EASI, eczema and disease severity index; EASI^high^, AD patients with high disease severity; EASI^low^, AD patients with low disease severity; Eosinophilia^high^, AD patients with high eosinophil counts in blood; Eosinophilia^low^, AD patients with low eosinophil counts in blood; Total IgE^high^, AD patients with high total IgE levels in plasma; Total IgE^low^, AD patients with low total IgE levels in plasma.

## Data Availability

The raw data supporting the conclusions of this article will be made available by the authors on request.

## Supplementary Materials

The following supporting information can be downloaded at: https://www.mdpi.com/article/10.3390/ijms26167821/s1.

## References

[1] Langan S.M., Irvine A.D., Weidinger S. (2020). Atopic dermatitis. Lancet.

[2] Guttman-Yassky E., Renert-Yuval Y., Brunner P.M. (2025). Atopic dermatitis. Lancet.

[3] Livesey A., Garty F., Shipman A.R., Shipman K.E. (2020). Lactate dehydrogenase in dermatology practice. Clin. Exp. Dermatol..

[4] Mukai H., Noguchi T., Kamimura K., Nishioka K., Nishiyama S. (1990). Significance of elevated serum LDH (lactate dehydrogenase) activity in atopic dermatitis. J. Dermatol..

[5] Renert-Yuval Y., Thyssen J.P., Bissonnette R., Bieber T., Kabashima K., Hijnen D., Guttman-Yassky E. (2021). Biomarkers in atopic dermatitis-a review on behalf of the International Eczema Council. J. Allergy Clin. Immunol..

[6] Simpson E.L., Villarreal M., Jepson B., Rafaels N., David G., Hanifin J., Taylor P., Boguniewicz M., Yoshida T., De Benedetto A. (2018). Patients with Atopic Dermatitis Colonized with Staphylococcus aureus Have a Distinct Phenotype and Endotype. J. Investig. Dermatol..

[7] Kou K., Aihara M., Matsunaga T., Chen H., Taguri M., Morita S., Fujita H., Yamaguchi Y., Kambara T., Ikezawa Z. (2012). Association of serum interleukin-18 and other biomarkers with disease severity in adults with atopic dermatitis. Arch. Dermatol. Res..

[8] Kato A., Kamata M., Ito M., Uchida H., Nagata M., Fukaya S., Hayashi K., Fukuyasu A., Tanaka T., Ishikawa T. (2020). Higher baseline serum lactate dehydrogenase level is associated with poor effectiveness of dupilumab in the long term in patients with atopic dermatitis. J. Dermatol..

[9] Czarnowicki T., Santamaria-Babí L.F., Guttman-Yassky E. (2017). Circulating CLA(+) T cells in atopic dermatitis and their possible role as peripheral biomarkers. Allergy.

[10] Bakker D.S., Nierkens S., Knol E.F., Giovannone B., Delemarre E.M., van der Schaft J., van Wijk F., de Bruin-Weller M.S., Drylewicz J., Thijs J.L. (2021). Confirmation of multiple endotypes in atopic dermatitis based on serum biomarkers. J. Allergy Clin. Immunol..

[11] Dekkers C., der Wal M.M.V., El Amrani M., Luin M.V., Bakker D.S., Bruin-Weller M., van Wijk F. (2023). Biological Tipping Point in Patients with Atopic Dermatitis Treated with Different Dosing Intervals of Dupilumab. J. Investig. Dermatol..

[12] Jack C., Allakhverdi Z., Dupuis F. 185 High-dimensional analysis identifies variability persistent skin-homing Th2/Tc2 populations in AD patients under remission with dupilumab. Proceedings of the ISID 2023.

[13] Starrenburg M., Dekkers C., van der Wal M., Meermans M., Bakker D., de Bruin-Weller M., van Wijk F. (2024). 121 Impact Of Tralokinumab on Skin-homing T cells and IL-4 and IL13 Receptor Dynamics In Patients With Atopic Dermatitis. J. Investig. Dermatol..

[14] Elsner J.S., Carlsson M., Stougaard J.K., Nygaard U., Buchner M., Fölster-Holst R., Hvid M., Vestergaard C., Deleuran M., Deleuran B. (2020). The OX40 Axis is Associated with Both Systemic and Local Involvement in Atopic Dermatitis. Acta Derm. Venereol..

[15] Weidinger S., Bieber T., Cork M.J., Reich A., Wilson R., Quaratino S., Stebegg M., Brennan N., Gilbert S., O′Malley J.T. (2023). Safety and efficacy of amlitelimab, a fully human nondepleting, noncytotoxic anti-OX40 ligand monoclonal antibody, in atopic dermatitis: Results of a phase IIa randomized placebo-controlled trial. Br. J. Dermatol..

[16] Sans-De San Nicolàs L., Figueras-Nart I., Bonfill-Ortí M., De Jesús-Gil C., García-Jiménez I., Guilabert A., Curto-Barredo L., Bertolín-Colilla M., Ferran M., Serra-Baldrich E. (2022). SEB-induced IL-13 production in CLA(+) memory T cells defines Th2 high and Th2 low responders in atopic dermatitis. Allergy.

[17] Sans-de San Nicolàs L., Figueras-Nart I., García-Jiménez I., Bonfill-Ortí M., Guilabert A., Curto-Barredo L., Bertolín-Colilla M., Ferran M., Serra-Baldrich E., Pujol R.M. (2023). Allergen sensitization stratifies IL-31 production by memory T cells in atopic dermatitis patients. Front. Immunol..

[18] García-Jiménez I., Sans-de San Nicolás L., Curto-Barredo L., Bertolín-Colilla M., Sensada-López E., Figueras-Nart I., Bonfill-Ortí M., Guilabert-Vidal A., Ryzhkova A., Ferran M. (2024). Heterogeneous IL-9 Production by Circulating Skin-Tropic and Extracutaneous Memory T Cells in Atopic Dermatitis Patients. Int. J. Mol. Sci..

[19] Campanati A., Bianchelli T., Gesuita R., Foti C., Malara G., Micali G., Amerio P., Rongioletti F., Corazza M., Patrizi A. (2022). Comorbidities and treatment patterns in adult patients with atopic dermatitis: Results from a nationwide multicenter study. Arch. Dermatol. Res..

[20] Tsuboi H., Kouda K., Takeuchi H., Takigawa M., Masamoto Y., Takeuchi M., Ochi H. (1998). 8-hydroxydeoxyguanosine in urine as an index of oxidative damage to DNA in the evaluation of atopic dermatitis. Br. J. Dermatol..

[21] Morishima Y., Kawashima H., Takekuma K., Hoshika A. (2010). Changes in serum lactate dehydrogenase activity in children with atopic dermatitis. Pediatr. Int..

[22] Tsoi L.C., Rodriguez E., Degenhardt F., Baurecht H., Wehkamp U., Volks N., Szymczak S., Swindell W.R., Sarkar M.K., Raja K. (2019). Atopic Dermatitis Is an IL-13-Dominant Disease with Greater Molecular Heterogeneity Compared to Psoriasis. J. Investig. Dermatol..

[23] Bilsborough J., Leung D.Y., Maurer M., Howell M., Boguniewicz M., Yao L., Storey H., LeCiel C., Harder B., Gross J.A. (2006). IL-31 is associated with cutaneous lymphocyte antigen-positive skin homing T cells in patients with atopic dermatitis. J. Allergy Clin. Immunol..

[24] Acevedo N., Benfeitas R., Katayama S., Bruhn S., Andersson A., Wikberg G., Lundeberg L., Lindvall J.M., Greco D., Kere J. (2020). Epigenetic alterations in skin homing CD4(+)CLA(+) T cells of atopic dermatitis patients. Sci. Rep..

[25] Czarnowicki T., Gonzalez J., Shemer A., Malajian D., Xu H., Zheng X., Khattri S., Gilleaudeau P., Sullivan-Whalen M., Suárez-Fariñas M. (2015). Severe atopic dermatitis is characterized by selective expansion of circulating TH2/TC2 and TH22/TC22, but not TH17/TC17, cells within the skin-homing T-cell population. J. Allergy Clin. Immunol..

[26] Bakker D.S., van der Wal M.M., Heeb L.E.M., Giovannone B., Asamoah M., Delemarre E.M., Drylewicz J., Nierkens S., Boyman O., de Bruin-Weller M.S. (2021). Early and Long-Term Effects of Dupilumab Treatment on Circulating T-Cell Functions in Patients with Moderate-to-Severe Atopic Dermatitis. J. Investig. Dermatol..

[27] Olesen C.M., Holm J.G., Nørreslet L.B., Serup J.V., Thomsen S.F., Agner T. (2019). Treatment of atopic dermatitis with dupilumab: Experience from a tertiary referral centre. J. Eur. Acad. Dermatol. Venereol..

[28] Lee Y., Kim M.E., Nahm D.H. (2021). Real Clinical Practice Data of Monthly Dupilumab Therapy in Adult Patients With Moderate-to-Severe Atopic Dermatitis: Clinical Efficacy and Predictive Markers for a Favorable Clinical Response. Allergy Asthma Immunol. Res..

[29] Makowska K., Nowaczyk J., Blicharz L., Waśkiel-Burnat A., Czuwara J., Olszewska M., Rudnicka L. (2023). Immunopathogenesis of Atopic Dermatitis: Focus on Interleukins as Disease Drivers and Therapeutic Targets for Novel Treatments. Int. J. Mol. Sci..

[30] Bieber T. (2020). Interleukin-13: Targeting an underestimated cytokine in atopic dermatitis. Allergy.

[31] Santamaria Babi L.F., Picker L.J., Perez Soler M.T., Drzimalla K., Flohr P., Blaser K., Hauser C. (1995). Circulating allergen-reactive T cells from patients with atopic dermatitis and allergic contact dermatitis express the skin-selective homing receptor, the cutaneous lymphocyte-associated antigen. J. Exp. Med..

[32] Trier A.M., Kim B.S. (2023). Insights into atopic dermatitis pathogenesis lead to newly approved systemic therapies. Br. J. Dermatol..

[33] Jura-Szoltys E., Niemiec-Górska A., Glück J., Branicka O., Gawlik R. (2025). Evaluation of Rhinological Symptoms and Quality of Life in Patients with Allergic or Eosinophilic Severe Uncontrolled Asthma Treated with Anti-IgE or Anti-IL5 Therapy: A Real-Live Study. Int. Arch. Allergy Immunol..

[34] Soendergaard M.B., Hansen S., Bjerrum A.S., Hilberg O., Lock-Johansson S., Håkansson K.E.J., Ingebrigtsen T.S., Johnsen C.R., Rasmussen L.M., von Bülow A. (2022). Complete response to anti-interleukin-5 biologics in a real-life setting: Results from the nationwide Danish Severe Asthma Register. ERJ Open Res..

[35] Dugas B., Renauld J.C., Pène J., Bonnefoy J.Y., Peti-Frère C., Braquet P., Bousquet J., Van Snick J., Mencia-Huerta J.M. (1993). Interleukin-9 potentiates the interleukin-4-induced immunoglobulin (IgG, IgM and IgE) production by normal human B lymphocytes. Eur. J. Immunol..

[36] Petit-Frere C., Dugas B., Braquet P., Mencia-Huerta J.M. (1993). Interleukin-9 potentiates the interleukin-4-induced IgE and IgG1 release from murine B lymphocytes. Immunology.

[37] Iyengar S.R., Hoyte E.G., Loza A., Bonaccorso S., Chiang D., Umetsu D.T., Nadeau K.C. (2013). Immunologic effects of omalizumab in children with severe refractory atopic dermatitis: A randomized, placebo-controlled clinical trial. Int. Arch. Allergy Immunol..

[38] Roesner L.M., Farag A.K., Pospich R., Traidl S., Werfel T. (2022). T-cell receptor sequencing specifies psoriasis as a systemic and atopic dermatitis as a skin-focused, allergen-driven disease. Allergy.

[39] Steinhoff M., Ahmad F., Pandey A., Datsi A., AlHammadi A., Al-Khawaga S., Al-Malki A., Meng J., Alam M., Buddenkotte J. (2022). Neuroimmune communication regulating pruritus in atopic dermatitis. J. Allergy Clin. Immunol..

[40] Lou H., Lu J., Choi E.B., Oh M.H., Jeong M., Barmettler S., Zhu Z., Zheng T. (2017). Expression of IL-22 in the Skin Causes Th2-Biased Immunity, Epidermal Barrier Dysfunction, and Pruritus via Stimulating Epithelial Th2 Cytokines and the GRP Pathway. J. Immunol..

[41] Siiskonen H., Harvima I. (2019). Mast Cells and Sensory Nerves Contribute to Neurogenic Inflammation and Pruritus in Chronic Skin Inflammation. Front. Cell. Neurosci..

[42] Beck L.A., Thaçi D., Hamilton J.D., Graham N.M., Bieber T., Rocklin R., Ming J.E., Ren H., Kao R., Simpson E. (2014). Dupilumab treatment in adults with moderate-to-severe atopic dermatitis. N. Engl. J. Med..

[43] Silverberg J.I., Pinter A., Alavi A., Lynde C., Bouaziz J.D., Wollenberg A., Murrell D.F., Alpizar S., Laquer V., Chaouche K. (2021). Nemolizumab is associated with a rapid improvement in atopic dermatitis signs and symptoms: Subpopulation (EASI ≥ 16) analysis of randomized phase 2B study. J. Eur. Acad. Dermatol. Venereol..

[44] Guttman-Yassky E., Brunner P.M., Neumann A.U., Khattri S., Pavel A.B., Malik K., Singer G.K., Baum D., Gilleaudeau P., Sullivan-Whalen M. (2018). Efficacy and safety of fezakinumab (an IL-22 monoclonal antibody) in adults with moderate-to-severe atopic dermatitis inadequately controlled by conventional treatments: A randomized, double-blind, phase 2a trial. J. Am. Acad. Dermatol..

[45] Thaçi D. IL-22 receptor blocker reduces itch and skin lesions in AD. Proceedings of the Ammerican Academy of Dermatology (AAD).

[46] Lu Z., Zeng N., Cheng Y., Chen Y., Li Y., Lu Q., Xia Q., Luo D. (2021). Atopic dermatitis and risk of autoimmune diseases: A systematic review and meta-analysis. Allergy Asthma Clin. Immunol..

[47] Ferran M., Galván A.B., Rincón C., Romeu E.R., Sacrista M., Barboza E., Giménez-Arnau A., Celada A., Pujol R.M., Santamaria-Babí L.F. (2013). Streptococcus induces circulating CLA(+) memory T-cell-dependent epidermal cell activation in psoriasis. J. Investig. Dermatol..

