# Supplementary material for: Patient Stratification for Serum LDH Levels Reveals Distinct CLA+ T-Cell Cytokine Secretion in Response to HDM, Clinical Features and Allergic Comorbidities
Source: Int J Mol Sci. 2025 Aug 13;26(16):7821. doi: 10.3390/ijms26167821 (PMC12387014; doi:10.3390/ijms26167821)
Supplement: Supplementary file 1 [file ijms-26-07821-s001.zip › ijms-3602569-supplementary.pdf]

SUPPORTING FIGURES

Figure S1. AD patients were stratified according to median values of (A) total IgE (n = 47), (B) blood eosinophil count (n = 46) and (C) EASI (n = 47). EASI, eczema and area score index.

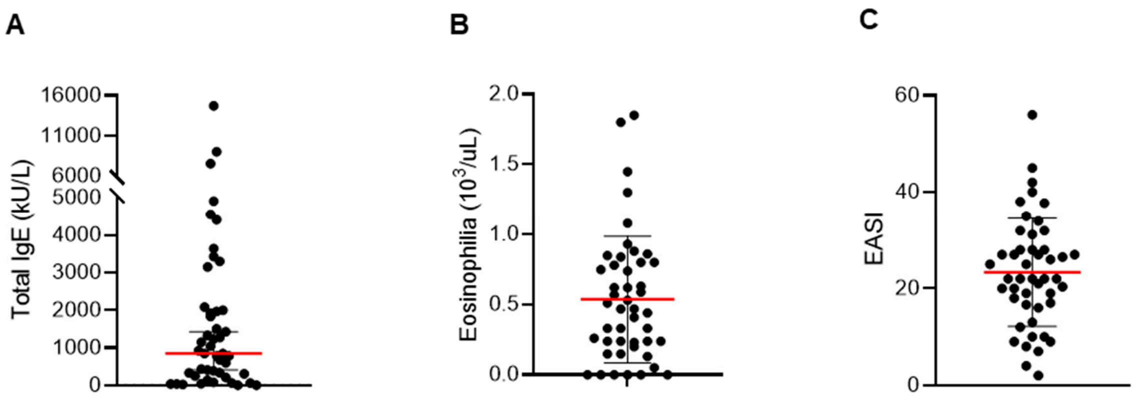

SUPPORTING TABLES

Table S1. Comparison of specific and total IgE levels between AD subgroups and control individuals.

				P value	
**Clinical features**	**LDH^{high}**	**LDH^{low}**	**Controls**	H vs C	L vs C
Total IgE (kU/L)	1666 (475.3-4220)	433 (40.7-926)	9.5 (1.9-21.1)	**<.0001**	**.0004**
HDM-sp IgE (OD)	17055 (6482-21464)	5338 (376-13441)	24.0 (22.0-49.0)	**<.0001**	**.0002**
SEB-sp IgE (kU/L)	0.3 (0.13-2.47)	0.13 (0.0-0.44)	0.0 (0.0-0.0)	**<.0001**	**.006**

Data is presented as medians (25th-75th percentiles). C, controls; H, LDH^{high} AD subgroup; HDM, house dust mite; L, LDH^{low} AD subgroup; OD, optical density; SEB, staphylococcal enterotoxin B; sp, specific. Bold values indicate significant data.

Table S2. Comparison of CLA+ T-cell-mediated cytokine responses between LDH^{high} and LDH^{low}, adjusted for disease severity (EASI).

Variable	MD (CI 95%)	p-value[1]	p-value[2]
IL13	52.3 (0.1-104.6)	**0.056**	0.150
IL4	18.1 (-2.1-38.3)	**0.085**	0.171
IL5	17.2 (5.7-28.7)	**0.005**	**0.043**
IL31	0 (-15.7-15.7)	1.000	1.000
IL17A	1.3 (-10.6-13.3)	0.826	1.000
IL22	0 (-38.4-38.4)	1.000	1.000
IFNγ	0 (-12.2-12.2)	1.000	1.000
IL9	181.8 (43.3-320.2)	**0.014**	**0.055**

Data is presented as mean differences with 95% confidence intervals.

[1] adjusted by EASI

[2] FDR correction applied

Table S3. Clinical features of AD patients and control individuals.

Clinical features	Patients with AD	N	Control individuals	N
Age	30.00 (24.00-45.00)	47	55.00 (37.00-69.00)	11
Female gender, n (%)	23 (48.94%)	47	4 (36.36)	11
Disease duration	20.00 (9.50-28.00)	46	–	–
Age at disease onset	7.00 (1.00-27.00)	46	–	–
EASI	22.0 (16.70-28.00)	47	–	–
IGA	3.00 (3.00-4.00)	47	–	–
Pruritus (VAS)	8.00 (7.00-9.00)	47	–	–
Eosinophilia (x103/µL)	0.47 (0.22-0.80)	46	–	–
Serum LDH (U/L)	206 (144-243)	47	152 (139-200)	11
Total IgE (kU/L)	844.90 (245-2000)	47	9.50 (1.90-21.10)	11
HDM-sp IgE (OD)	13213 (2854-18962)	47	24.00 (22.00-49.00)	11
SEB-sp IgE (kU/L)	0.17 (0.00-0.84)	46	0.00 (0.00-0.00)	11
Allergic rhinitis, n (%)	27 (58.70%)	47	–	–
Allergic Asthma, n (%)	21 (45.65%)	47	–	–
Allergic Conjunctivitis, n (%)	18 (39.13%)	47	–	–
Food Allergy, n (%)	10 (22.73%)	45	–	–

Catergorical variables are presented as counts (percentages) and numerical variables are presented as medians (25th-75th percentiles). AD, atopic dermatitis; EASI, eczema area and severity index; HDM, house dust mite; IGA, investigator's global assessment; OD, optical density; SEB, staphylococcal enterotoxin B; sp, specific; VAS, visual analogue scale.

SUPPORTING MATERIALS AND METHODS

To evaluate the potential confounding effect of disease severity (EASI score) on the association between LDH status and Th2 cytokine expression in the CLA^{+} T-cell compartment, we performed median regression (quantile regression at $\tau = 0.5$) for eight HDM-stimulated cytokines: IL-13, IL-4, IL-5, IL-31, IL-17A, IL-22, IFN-γ, and IL-9. For each cytokine, two models were fitted: one with LDH status (high vs. low) as predictor, and a second model adjusting for EASI score to account for disease severity. Quantile regression was implemented using the rq function from the *quantreg* R package (v5.97), with standard errors and confidence intervals estimated via bootstrapping (R = 100) using the "xy" method. Estimated marginal effects for the LDH^{high} group were reported with 95% confidence intervals and both nominal and FDR-adjusted p-values (Benjamini-Hochberg correction).
